# Incidence and risk factors of postoperative hyperamylasemia and pancreatitis following total knee arthroplasty: a retrospective study

**DOI:** 10.1186/s12891-023-06714-1

**Published:** 2023-07-17

**Authors:** Song Liu, Fangguo Li, Wei Hu, Qihao Yang, Chi Zhang, Zhao Wang

**Affiliations:** 1 Department of Orthopaedic Surgery, The Third Affiliated Hospital of Guangzhou Medical University, Guangzhou, 510150 P. R. China; 2 Department of Orthopaedics, Tianjin Hospital, Tianjin, P. R. China

**Keywords:** Postoperative hyperamylasemia, Postoperative pancreatitis, Complication, Total knee arthroplasty

## Abstract

**Background:**

Postoperative hyperamylasemia and pancreatitis are recognized complications after abdominal and spinal surgeries. The aim of this study is to investigate the incidence and identify risk factors for postoperative hyperamylasemia and pancreatitis following total knee arthroplasty.

**Methods:**

170 patients undergoing total knee arthroplasty were retrospectively identified from our database from January 2017 to January 2021. Patients were divided into normal and hyperamylasemia groups based on the presence of serum amylase level within or greater than the normal range. The diagnosis of postoperative pancreatitis was based on the 2012 revised Atlanta Classification of Acute Pancreatitis. Patient demographics, perioperative parameters were investigated with student t test, chi square test and multivariate logistic regression analysis.

**Results:**

43 patients (25.3%) exhibited postoperative hyperamylasemia while eight patients (4.7%) exhibited serum amylase < 5 times the normal upper limit. One patient (0.6%) was designated as having postoperative pancreatitis. More patients with Hypertriglyceridemia (HTG) were noted in hyperamylasemia group (*P* = 0.009) compared with normal group. Hyperamylasemia group showed higher preoperative serum amylase (74.95 vs. 55.62 IU/L, *P* < 0.001), higher intra-operative blood loss (IBL) (117.67 vs. 77.01 mL, *P* = 0.040) and longer surgical duration (132.98 vs. 107.01 min, *P* = 0.041). Multivariate logistic analysis revealed that HTG (OR = 0.189, P = 0.006), preoperative serum amylase (OR = 1.042, *P* < 0.001) and IBL (OR = 1.004, *P* = 0.022) were independent risk factors for postoperative hyperamylasemia.

**Conclusions:**

A significant percentage of patients developed hyperamylasemia after total knee arthroplasty. Patients with HTG, higher preoperative serum amylase and higher IBL had an increased risk of developing postoperative hyperamylasemia and pancreatitis.

## Background

Postoperative pancreatitis has been described as a serious complication of various abdominal or nonabdominal surgeries, such as cardiac, parathyroid, and prostate surgeries [1–4]. In the field of spinal surgery, it has been reported that postoperative hyperamylasemia and pancreatitis are rare but detrimental complications following posterior spinal surgery in adults or in children and young adults with or without cerebral palsy who have scoliosis [5–10]. However, postoperative hyperamylasemia and pancreatitis following joint arthroplasty are rarely noticed and thus often neglected. Saah, R.B. et al. [11] reported a case of postoperative pancreatitis after simultaneous bilateral total knee arthroplasty (TKA), although this patient only presented persistent tachycardia, without abdominal pain or nausea. Szymanski, et al. [12] reported another case of postoperative pancreatitis after bilateral TKA, the authors discussed the association of postoperative pancreatitis with a delayed hemolytic transfusion reaction. Passaglia, J. et al. [13] recently identified gallstones/pancreatitis as one of the gastrointestinal complications following total joint arthroplasty. Connor et al. [14] reported a case of acute pancreatitis (AP) following high tibial osteotomy, which indicated that obesity may be a potential risk factor. Despite the above studies, there remains limited knowledge on the incidence and consequence of postoperative hyperamylasemia and pancreatitis following joint arthroplasty. It is acknowledged that hyperamylasemia can be identified in pancreatic or salivary gland-related conditions such as AP, trauma, alcoholism, amylase-producing tumor, while pancreatitis can be caused mainly in pancreatic conditions such as gastroduodenal surgery, alcoholism, bile stones, exacerbation of chronic pancreatitis [15, 16]. However, the risk factors and etiology of postoperative hyperamylasemia and pancreatitis following TKA remains unknown.

Our hospital is in liwan district, Guangzhou, Guangdong Province. The history of this district can be dated back to the initial development of Guangzhou and thus many elderlies reside in this area. The latest national census in 2021 reveals that approximately 19.8% of the population in liwan district ages over 60 years old and 13.7% ages over 65. With the gradual aging of population, the number of patients with osteoporosis and joint degeneration is seeing an upward trend year by year. Given the aging Chinese population and the inevitable degenerative process of the knee, a substantial increase in the overall volume of joint arthroplasties is expected in China. Globally, it has been projected that the total annual number of total joint arthroplasty of the hip or knee procedures in US will exceed four million by 2030 [17]. Siviero. et al. [18] have recently conducted a prospective observational study showing that TKA significantly improves quality of life and physical function in patients with end-stage OA 3-months after surgery and remains to be a valid surgical approach in severe OA patients. Therefore, a comprehensive perioperative evaluation and management is essential to avoid serious complications and achieve satisfactory outcome in patients who undergo joint arthroplasty. This study aims to investigate the incidence of postoperative hyperamylasemia and pancreatitis following TKA and to identify potential risk factors that may predict the incidence of postoperative hyperamylasemia and pancreatitis.

## Methods

This study was approved by the institutional review board of the Third Affiliated Hospital of Guangzhou Medical University and all methods were carried out in accordance with the Declaration of Helsinki. The need for informed consent was waived by the institutional review board of the Third Affiliated Hospital of Guangzhou Medical University. A total of 765 patients underwent TKA were performed in our institution from January 2017 to January 2021. However, only 170 patients were included, the others were excluded because serum amylase was not measured. The normal range for serum amylase is 25-125IU/L in our institute. Based on the criteria of 2012 revised Atlanta Classification of Acute Pancreatitis [19], the diagnosis of postoperative pancreatitis requires two of the following three features: (1) abdominal pain consistent with acute pancreatitis; (2) at least three times increase in serum lipase level or serum amylase level compared with the normal upper limit; and (3) characteristic findings of acute pancreatitis on contrast-enhanced computed tomography (CECT), magnetic resonance imaging or transabdominal ultrasonography. Demographic data obtained included the patient’s age, gender, height, weight, and body mass index (BMI). Medical comorbidities obtained included hypertension, Hypertriglyceridemia (HTG), diabetes, digestive disease, pancreatic disease, liver disease and alcohol abuse. Results of routine blood test and biochemistry blood test obtained included C-reactive protein (CRP), white blood cell (WBC) count, absolute neutrophil count (ANC), neutrophil percentage, absolute lymphocyte count (ALC), lymphocyte percentage, absolute monocyte count (AMC), monocyte percentage, absolute eosinophil count (AEC), eosinophil percentage, hemoglobin (HGB), hematocrit (HCT), albumin/globulin (A/G) ratio, alanine aminotransferase (ALT), aspartate aminotransferase (AST) and creatinine (Cre) pre- or postoperatively. Perioperative factors obtained included estimated blood volume (EBV), total blood loss (TBL), postoperative blood loss (PBL), intra-operative blood loss (IBL), allogeneic red blood cell (RBC) transfusion, and surgical duration. PBL equals to the postoperative drainage at postoperative day 1 (POD1). EBV and TBL were calculated based on the following Equations [20–22]:

EBV (L) = k1 ∗ height (m)^3^ + k2 ∗ weight (kg) + k3.

TBL (L) = EBV (L) * (Hct_pre_ - Hct_post_).

where k1 = 0.3669, k2 = 0.03219, and k3 = 0.6041 for males, and k1 = 0.3561, k2 = 0.03308, and k3 = 0.1833 for females.

### Statistical analysis

All data analysis were performed using SPSS version 25.0. The continuous variables were measured as the mean ± standard deviation, and the categorical variables were measured as a percentage or number of cases. Student t test and Chi square test were used to assess the statistical significance between the hyperamylasemia group and normal group in demographic statistics, preoperative factors, perioperative factors, postoperative factors, surgical procedures, and comorbidities, with a significance level of P < 0.05. Multivariate logistic regression analysis was then conducted for factors with P < 0.05 in the student t test or chi square test. Odds ratios (ORs) and their 95% confidence interval (CI) were estimated with multivariate logistic regression models to evaluate the association of hyperamylasemia with those variables which have been screened out. The variables those were screened out by multivariate logistic regression model were assessed by the receiver operating characteristic (ROC) curves. The continuous variables were divided into different subgroups according to the cut-off values. Cut-off values, sensibility and specificity were decided according to the assessment of ROC curves.

## Results

Over a four-year period, 765 patients underwent TKA in our institution, of which 170 patients who met the criteria were included. 43 patients (25.3%) had increased serum amylase on POD1.

No significant difference was found between hyperamylasemia and normal groups in terms of patient demographics. Comparing the comorbidities between the groups, more patients with HTG were noted in hyperamylasemia group (P = 0.009) (Table 1). Patients in hyperamylasemia group had significantly higher preoperative serum amylase (74.95 vs. 55.62 IU/L, P < 0.001) preoperatively (Table 2). Patients in hyperamylasemia group had significantly higher IBL (117.67 vs. 77.01 mL, P = 0.040) and longer surgical duration (132.98 vs. 107.01 min, P = 0.041) on POD1 (Table 3).

**Table 1:** Demographics and comorbidities in groups of patients with hyperamylasemia and with normal serum amylase levels

	Hyperamylasemia (n = 43)	Normal (n = 127)	P-Value
**Demographics**			
Age (years)	68.84 (7.62)	69.87 (8.65)	0.489
Height (m)	1.57 (0.06)	1.59 (0.07)	0.325
Weight (kg)	63.51 (7.30)	65.15 (11.02)	0.381
BMI (kg/m^2^) Gender (men)	25.65 (3.15) 3 (3/43)	25.80 (3.78) 25 (25/127)	0.825 0.052
**Comorbidities**			
Hypertension HTG Diabetes Digestive disease	26 (26/43) 8 (8/43) 4 (4/43) 3 (3/43)	81 (81/127) 7 (7/127) 26 (26/127) 5 (5/127)	0.697 0.009 0.097 0.416
Pancreatic disease	0(0/43)	1(1/127)	0.569
Liver disease	2(2/43)	8(8/127)	0.982
Alcohol abuse	1(1/43)	7(7/127)	0.663

*BMI*, body mass index; *HTG*, hypertriglyceridemia

**Table 2:** Preoperative and postoperative laboratory parameters in groups of patients with hyperamylasemia and with normal serum amylase levels

	Hyperamylasemia (n = 43)	Normal (n = 127)	P-Value
**Preoperative parameters**			
CRP (mg/L)	7.86 (11.97)	9.18 (20.61)	0.703
WBC (10^9^/L)	6.99 (1.57)	6.97 (1.51)	0.375
ANC (10^9^/L)	4.18 (1.17)	4.45 (1.81)	0.376
Neutrophil percentage (%)	60.30 (8.66)	61.33 (10.29)	0.561
ALC (10^9^/L)	2.04 (0.72)	1.88 (0.66)	0.198
Lymphocyte percentage (%)	29.38 (7.71)	27.87 (9.53)	0.367
AMC (10^9^/L)	0.44 (0.13)	0.46 (0.15)	0.465
Monocyte percentage (%)	6.98 (4.56)	6.69 (1.58)	0.540
AEC (10^9^/L)	0.25 (0.15)	0.22 (0.18)	0.409
Eosinophil percentage (%)	3.45 (1.87)	3.18 (2.69)	0.563
Serum amylase (IU/L)	74.95 (25.46)	55.62 (19.70)	< 0.001
HGB (g/L)	124.91 (18.45)	124.89 (16.37)	0.995
HCT (%)	38.17 (4.84)	38.12 (4.74)	0.956
A/G ratio	1.34 (0.25)	1.28 (0.24)	0.184
ALT (IU/L)	14.47 (5.23)	17.95 (11.93)	0.057
AST (IU/L)	16.36 (3.54)	20.97 (18.08)	0.132
Cre (µmol/L)	69.61 (21.47)	74.02 (21.29)	0.252
**Postoperative parameters**			
CRP (mg/L)	32.78 (35.11)	35.02 (47.78)	0.785
WBC (10^9^/L)	12.76 (2.99)	12.53 (3.42)	0.700
ANC (10^9^/L)	11.07 (2.97)	11.03 (3.92)	0.949
Neutrophil percentage (%)	86.14 (6.16)	85.69 (7.25)	0.718
ALC (10^9^/L)	1.09 (0.43)	1.11 (0.51)	0.772
Lymphocyte percentage (%)	8.88 (3.94)	9.87 (7.00)	0.394
AMC (10^9^/L)	0.57 (0.30)	0.56 (0.31)	0.796
Monocyte percentage (%)	4.60 (2.41)	4.57 (2.51)	0.945
AEC (10^9^/L)	0.03 (0.08)	0.03 (0.09)	0.898
Eosinophil percentage (%)	0.33 (0.85)	0.35 (0.92)	0.918
HGB (g/L)	108.84 (16.40)	113.56 (14.17)	0.072
HCT (%)	33.00 (4.61)	34.48 (4.34)	0.058
A/G ratio	1.31 (0.26)	1.22 (0.24)	0.459
ALT (IU/L)	13.57 (12.19)	16.40 (10.29)	0.139
AST (IU/L)	18.08 (8.03)	18.92 (6.99)	0.554
Cre (µmol/L)	70.95 (20.33)	76.96 (27.34)	0.198

*CRP*, C-reactive protein; *WBC*, white blood cell count; *ANC*, absolute neutrophil count; *ALC*, absolute lymphocyte count; *AMC*, absolute monocyte count; *AEC*, absolute eosinophil count; *HGB*, hemoglobin; *HCT*, hematocrit; *A/G*, albumin/globulin ratio; *ALT*, alanine aminotransferase; *AST*, aspartate aminotransferase; *Cre*, creatinine

**Table 3:** Intraoperative details, length of stay and inpatient cost in groups of patients with hyperamylasemia and with normal serum amylase levels

	Hyperamylasemia (n = 43)	Normal (n = 127)	P-Value
EBV (L)	3.71 (0.76)	3.85 (0.60)	0.138
TBV (mL)	164.72 (143.72)	132.84 (141.34)	0.219
PBL (mL)	122.53 (82.72)	117.32 (84.25)	0.725
IBL (mL)	117.67 (152.14)	77.01 (93.66)	0.040
Allogeneic RBC transfusion	3 (3/43)	3 (3/127)	0.171
Surgical duration (min)	132.98 (34.75)	107.01 (30.37)	0.041
Length of stay (day)	16.34 (6.21)	15.13 (5.53)	0.232
Inpatient cost (RMB)	78646.48 (20357.76)	74343.06 (24237.06)	0.297

*EBV*, estimated blood volume; *TBL*, total blood loss; *PBL*, postoperative blood loss; *IBL*, intra-operative blood loss

**Fig. 1:**
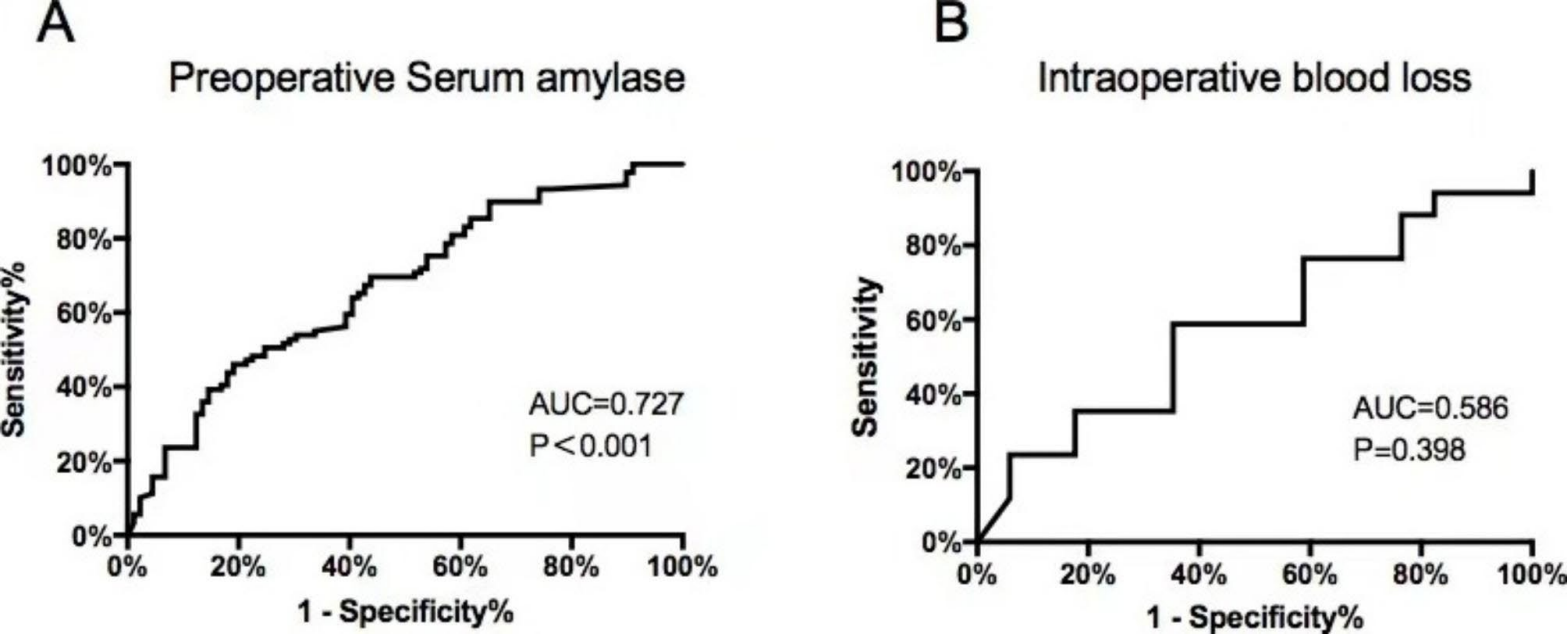
ROC of **(A)** preoperative serum amylase and **(B)** IBL. ROC, receiver operating characteristic curve. AUC, area under curve

Multivariate logistic regression showed that HTG (p = 0.006; OR, 0.189; 95% CI 0.058 to 0.615), preoperative serum amylase (P < 0.001; OR, 1.042; 95% CI 1.023 to 1.061) and IBL (P = 0.022; OR, 1.004; 95% CI, 1.001 to 1.008) were strongly associated with postoperative hyperamylasemia (Table 4). Preoperative Serum amylase and IBL predicted postoperative hyperamylasemia with an area under the receiver operating characteristic curve of 0.727 (P < 0.001; 95% CI: 0.637–0.817) (Fig. 1A) and 0.586 (P = 0.398; 95% CI: 0.485–0.687) (Fig. 1B). With preoperative Serum amylase, a cutoff point of 63.45 offered a sensitivity of 65.1% and a specificity of 75.6%.

**Table 4:** Multivariate Logistic Regression Analysis for the Occurrence of the hyperamylasemia

Risk factors	*β*	Wald X^2^	P-Value	Odds Ratio(95% Confidence interval)
Hypertriglyceridemia	-1.665	7.657	0.006	0.189 (0.058 ~ 0.615)
Preoperative Serum amylase	0.041	19.701	<0.001	1.042 (1.023 ~ 1.061)
IBL	0.004	5.242	0.022	1.004 (1.001 ~ 1.008)

There were eight patients (4.7%) with serum amylase level > five times the normal upper limit. The characteristics of these patients are shown in Table 5, including their mean preoperative serum amylase of 90.88 IU/L, mean surgical duration of 155.88 min. The serum amylase level was > 1000 IU/L in three cases (1.8%). Abdominal pain occurred in one case (0.6%).

**Table 5:** Summary of data for 8 patients with postoperative serum amylase levels ≥ 5 times the upper limit of normal serum values

Age/sex	operation	Comorbidities	Preoperative serum amylase (IU/L)	Postoperative serum amylase (IU/L)	Surgical duration (min)	Post-op digestive symptoms
79/F	TKA	HTG	97	1114	115	
76/F	TKA	HT/Diabetes	94	2370	200	
79/F	TKA	HT	95	879	112	
65/F	TKA	HT	86	883	142	
61/F 61/F 63/F	TKA TKA TKA	- HT -	98 58 113	1332 985 701	142 269 152	Abdominal pain
65/F	TKA	HTG	86	849	115	

*TKA*, total knee arthroplasty; *HTG*, hypertriglyceridemia; *HT*, hypertension

**Table 6:** Previous studies related to hyperamylasemia, pancreatitis, and spine or joint surgery

Authors	Year	Number of patients	Average age(year)	Surgical procedure	Surgical duration (min)	Hyperamylasemia (> 125 IU/L)	Hyperamylasemia (> 375 IU/L)	Clinical pancreatitis	Risk factors	Perioperative mortality
Leichtner et al.	1991	44	15	Posterior or anteroposterior fusion	330	10 (22.7%)	NA	4 (9.1%)	IBL	NA
Laplaza et al.	2002	80	14	Posterior, anterior, or anteroposterior fusion	NA	NA	NA	7 (8.8%)	Age/height/BMI	NA
Tsirikos et al.	2003	45	15	One-Stage or Two-Stage anteroposterior spinal fusion	NA	NA	NA	4 (8.9%)	NA	2
Borkhuu et al.	2009	355	14	Posterior or anteroposterior fusion	NA	NA	NA	109 (30.7%)	Gastrointestinal tube/reactive airway disease	1
Kobayashi et al.	2015	262	53	Posterior fusion or laminectomy	324	92 (35.1%)	6 (2.3%)	1 (0.4%)	Estimated blood loss/surgical duration	NA
Bouyousfi et al.	2016	571	16	Posterior, anterior, or anteroposterior fusion	NA	NA	NA	14 (2.5%)	NA	NA
Samdani et al.	2016	127	14	Posterior, anterior, or anteroposterior fusion	409	NA	NA	22 (17.3%)	NA	NA
Bendon et al.	2016	19	14	Posterior, anterior, or anteroposterior fusion	NA	NA	NA	1 (5.3%)	NA	1
Jalanko et al.	2018	91	15	Posterior, anteroposterior fusion	426	NA	NA	0	NA	NA
Abousamra et al.	2018	300	15	Posterior, anteroposterior fusion	390	NA	18 (6%)	10 (3.3%)	Gastrostomy dependence	NA
Verhofste et al.	2021	425	14	Posterior, anterior, or anteroposterior fusion	NA	NA	45 (10.6%)	33 (7.8%)	NA	NA
Massaglia et al.	2021	17,402	64	TJA	99.5	NA	NA	7 (0.04%)	NA	12
Present study	2021	170	69	TKA	173.5	43 (25.29%)	8 (4.71%)	1 (0.6%)	Preoperative serum amylase/IBL/HTG	NA

*IBL: intra-operative blood loss; BMI: body mass index; TJA*, total joint arthroplasty; *TKA*, total knee arthroplasty; HTG: Hypertriglyceridemia

## Discussion

To our knowledge, this is the first study reporting the incidence of postoperative hyperamylasemia and pancreatitis following total knee arthroplasty in adults. Approximately 30–60% of patients following cardiac surgery and 23–35% of patients following spinal surgeries exhibited hyperamylasemia [7, 8, 23, 24]. In this study, hyperamylasemia was detected in 25.3% (43/170) of patients following total knee arthroplasty, which falls within ranges mentioned above. Previous studies reported that 2.3–10.6% of patients following various spinal surgeries exhibited serum amylase > 5 times the normal upper limit [7–10]. In our study, it was detected in 4.7% (8/170) of patients. However, significantly elevated serum amylase does not always cause clinical symptoms. Among these eight patients, one patient presented with abdominal pain. Therefore, 0.6% of patients (1/170) met the criteria for postoperative pancreatitis in this study. Previous studies reported that 0.04–30.7% of patients following spinal surgeries and joint arthroplasty exhibited postoperative pancreatitis [7–10, 13, 24–29].

It is well established that HTG is closely associated with AP and is the third most common cause for AP following gallstones and alcohol abuse. In China, there is an increasing trend of hypertriglyceridemia induced acute pancreatitis (HTGAP). Researchers from Peking Union Medical College conducted an epidemiology study of 475 patients presented with AP from 2001 to 2016, HTGAP increased annually from 14.0 to 34.0% while gallstone-associated pancreatitis decreased annually from 42.9 to 32.2% [30]. In our study, HTG was firstly identified as an independent risk factor for postoperative hyperamylasemia following TKA. Our results showed that 18.6% (8/43) of patients in hyperamylasemia group suffered from HTG while only 5.5% (7/127) of patients in normal group suffered from HTG. The pathophysiology of HTGAP is associated with accumulation of free fatty acids and activation of the inflammatory response. However, free fatty acids level was not tested in this study, and no significant difference was found in any single biomarker for inflammation preoperatively or postoperatively.

In the current study, preoperative serum amylase was identified as another independent risk factor for postoperative hyperamylasemia. No previous studies have linked preoperative serum amylase with the incidence of postoperative hyperamylasemia probably because serum amylase is not routinely measured in the blood tests during perioperative period of arthroplasty (Table 6). Amylase is one of the digestive enzymes that is mainly secreted from the pancreas and salivary glands and is cleared by the mononuclear phagocyte system (MPS) and kidney. It is likely that a weak function of MPS or kidney could result in an accumulation of amylase in serum. However, no significant difference was found between hyperamylasemia and normal groups in AMC, monocyte percentage and serum creatinine preoperatively or postoperatively in this study. Additionally, previous studies [31, 32]reported that hyperamylasemia was associated with the peripheral blood eosinophil level. Kimura et al. [32] found that there was a significant negative correlation between increase in serum amylase and decrease in AEC in oral food challenge-positive patients with immediate food allergy. Weir et al. [31] reported that the change of % eosinophils was associated with the change of serum amylase and lipase in a pancreatic transplant recipient, and the authors suggested that peripheral eosinophilia may be a useful early indicator of pancreas graft rejection preceding changes in serum pancreatic enzymes. However, no significant difference was found in AEC and eosinophil percentage preoperatively or postoperatively in this study. Further prospective studies are needed to elucidate the etiology of this association between preoperative serum amylase and postoperative hyperamylasemia.

Another important finding of this study was that surgical duration was significantly increased in patients with postoperative hyperamylasemia compared to those with normal amylase. In agreement with our result, Kobayashi et al. [7] and Passaglia et al. [13] also linked longer surgical duration to a higher risk of developing postoperative hyperamylasemia and gastrointestinal complications which include postoperative pancreatitis following spinal surgery or total joint arthroplasty in adults. Interestingly, when comparing the incidence of postoperative pancreatitis between Passaglia’s study and the present study, shorter surgical duration resulted in a lower incidence of postoperative pancreatitis which further indicates the correlation between surgical duration and postoperative pancreatitis. However, others [9, 24, 28, 33] found no significant difference regarding surgical duration following spinal surgeries in children and young adults. This discrepancy might be because of the physiological differences between adults and children and further research is needed. Nevertheless, a longer surgical duration can result in an increased IBL, and a significantly increased IBL was also observed in patients in hyperamylasemia group within our study. Previous studies [7, 24, 34, 35] suggested that IBL can cause pancreatic ischemia due to reduced blood supply of the pancreas and thus lead to pancreatic injury and subsequent pancreatitis. Additionally, excessive IBL is among the most frequent causes of hypovolemia which treatment requires fluid resuscitation. It is acknowledged that hydroxyethyl starch (HES) is a synthetic colloid that is commonly used in fluid resuscitation due to its ability to expand blood volume. Previous studies [36, 37] reported that hyperamylasemia was associated with the administration of HES. They suggested that hyperamylasemia was induced by HES through the formation of an HES-amylase complex with delayed elimination by the kidney because of its size. However, no significant difference was found between hyperamylasemia and normal groups in terms of the administration of HES in some patients whose anesthesia records were available (data not shown).

Treatment of symptomatic pancreatitis is largely conservative, with disease severity and the presence of organ failure determining its prognosis. Therefore, early disease recognition and intensive treatment is of utmost importance [38]. It is acknowledged that CT is highly accurate and sensitive in diagnosing and demonstrating the extent of potential pancreatic injury. In our study, CT scan should be performed when serum amylase level > 5 times the normal limit was found, even if no clear clinical signs was observed. Luckily, no severe pancreatitis case was observed in this study. Although one patient presented with abdominal symptoms and underwent abdominal CT scan, she quickly recovered after receiving total parenteral nutrition, administration of proton-pump inhibitor and somatostatin. According to the medical record of this patient, she was diagnosed with bilateral knee osteoarthritis and osteoporosis. There was no history of any potential causes of pancreatitis, such as gallstones, alcoholism, or hypertriglyceridemia. No perioperative mortality was observed in this study and only few cases of postoperative pancreatitis related death after spinal surgery were reported in the literature. Burkhouse et al. [8] reported that one patient developed a severe hemorrhagic pancreatitis 48 h after a spine fusion and died. Bandon et al. [26] reported that one patient developed postoperative pancreatitis and died of multiorgan failure on the 30th postoperative day after an anterior release. Passaglia et al. [13] reported that total joint arthroplasty patients with gastrointestinal complications which included pancreatitis had greater in-hospital mortality rates.

The present study had several limitations. First, the number of patients enrolled in this study was small because this was a single-center retrospective study. It is possible that the sample size may influence our results. For example, AUC for preoperative serum amylase is only 0.727 indicating a weak predictive capability. Second, serum amylase was not planned and assessed in all cases. There are three different types of biochemistry blood tests available in our institution according to the scope of examination and only one of them contains serum amylase. Theoretically serum amylase should be ordered for patients who reports comorbidities such as pancreatic disease, liver disease or alcohol abuse. Third, the etiology of postoperative hyperamylasemia and pancreatitis is multifactorial and remains unclear. From the data of current study, it was unable to identify the cause of elevated preoperative serum amylase and differentiate two isoforms, pancreatic amylase, and salivary amylase. Indeed, previous studies [39, 40] showed that hyperamylasemia after cervical, mediastinal exploration, and major abdominal surgery mostly consisted of an increase in serum salivary amylase.

## Conclusions

This study found that 25.3% of patients developed postoperative hyperamylasemia and 0.6% of patients developed postoperative pancreatitis after total knee arthroplasty. Although this patient developed postoperative pancreatitis, her symptoms were mild, and no perioperative mortality was observed. HTG, preoperative serum amylase, and IBL were identified as independent risk factors for postoperative hyperamylasemia. Prospective studies are needed to confirm these findings and elucidate the etiology and long-term consequences of postoperative hyperamylasemia and pancreatitis following TKA.

## Data Availability

Data may be available from the corresponding author on reasonable request.

## Abbreviations

TKA: Total knee arthroplasty
AP: Acute pancreatitis
BMI: Body mass index
HTG: Hypertriglyceridemia
CRP: C-reactive protein
WBC: White blood cell count
ANC: Absolute neutrophil count
ALC: Absolute lymphocyte count
AMC: Absolute monocyte count
AEC: Absolute eosinophil count
HGB: Hemoglobin
HCT: Hematocrit
A/G: Albumin/globulin ratio
ALT: Alanine aminotransferase
AST: Aspartate aminotransferase
Cre: Creatinine
EBV: Estimated blood volume
TBL: Total blood loss
PBL: Postoperative blood loss
IBL: Intra-operative blood loss (IBL)
POD1: Postoperative day 1
ORs: Odds ratios
CI: Confidence interval
ROC: Receiver operating characteristic
HTGAP: Hypertriglyceridemia induced acute pancreatitis
MPS: Mononuclear phagocyte system
HES: Hydroxyethyl starch
